# EEG and seizures of pertussis encephalopathy in infants

**DOI:** 10.3389/fped.2026.1679807

**Published:** 2026-03-20

**Authors:** Xiaoqing Luo, Guangtao Kuang, Jun Jiang, Peipei Wei, Xin Shu

**Affiliations:** Department of Electrophysiology, Wuhan Children's Hospital, Tongji Medical College, Huazhong University of Science & Technology, Wuhan, China

**Keywords:** EEG, encephalopathy, infants, pertussis, seizures

## Abstract

**Objective:**

There are fewer reports on the electroencephalogram(EEG) and seizures of infants with pertussis encephalopathy. Our study retrospectively analyzed the EEG and seizures to raise awareness of pertussis encephalopathy.

**Methods:**

A total of six cases of infants with pertussis encephalopathy were identified, including 31 consecutive EEG monitoring data sets.

**Results:**

All infants with pertussis encephalopathy were age of onset ranging from 1 month and 16 days to 3 months and 13 days. Generalized Rhythmic Delta Activity (GRDA) was seen in 5 cases. Multifocal sharp waves and sharp-slow waves were observed in 6 cases, with triphasic sharp waves or sharp-slow waves in the temporal region in 4 cases. Theta rhythms were observed in 4 cases, showing temporal or temporally prominent activity. Four cases exhibited both electroclinical and electrographic seizures(ESzs), while two cases exhibited only electroclinical seizures (ECSzs). Seizure onset involved 3 or more regions in 3 cases, including the frontal, central, parietal, and temporal regions. Seizures were migratory in 4 out of 6 cases. ECSzs included focal clonic, behavioral arrest, and automatism in 2 cases, behavioral arrest in 2 cases, focal clonic in 1 cases, and focal clonic with behavioral arrest in 1 case. Seizures lasting more than 5 min were observed in 5 cases during serial EEG monitoring. When the white blood cell count dropped significantly or approached normal levels, seven or more ECSzs and ESzs were still recorded in 4 cases during four 2–4 h of EEG monitoring. Over a follow-up of 9 months to 2 years 5 months, no seizures recurred. Neurodevelopmental outcomes spanned a spectrum from normal (4 cases) to isolated slightly motor delay (Case 5) to significant global delay (Case 6).

**Conclusion:**

Infantile pertussis encephalopathy was characterized by ESzs and ECSzs, and notably, the severity of the seizure burden may not correlate with fluctuations in WBC count. These seizures were focal and may have a multifocal or migratory onset, manifesting as focal clonic seizures, behavioral arrest, or automatisms, with some seizures lasting more than 5 min. EEG abnormalities may included GRDA, multifocal (sometimes triphasic) sharp/sharp-slow waves, and theta rhythms that were prominent in the temporal regions. Aggressive treatment may achieve seizure freedom, but long-term neurodevelopmental outcomes remained heterogeneous.

## 1 Introduction

Pertussis, also known as whooping cough, is an acute respiratory infection caused by *Bordetella pertussis (*1, 2). Typical symptoms include paroxysmal coughing, whooping-like inhalation, and prolonged coughing episodes (3). Pertussis is a disease that can be prevented through vaccination (4). However, even in countries with high coverage of pertussis vaccination, the incidence of pertussis has risen again after years of remaining low, with some areas experiencing outbreaks (5). After the introduction of immunization programs, the transmission pattern has shifted from a previous child-to-child epidemic model to an adult-to-child epidemic model, making infants and young children more susceptible to infection (6, 7). According to estimates by the World Health Organization, there are approximately 24.1 million cases of pertussis globally each year, with about 160,700 deaths among children under the age of five (8). Due to their immature immune systems, infants are more likely to develop severe cases of pertussis, often requiring intensive care. Severe pertussis in infants can lead to encephalopathy, manifesting as symptoms such as lethargy, drowsiness, and seizures, and in severe cases, may result in long-term neurological sequelae (9). Early identification and treatment of pertussis encephalopathy are crucial for improving prognosis. Electroencephalography (EEG) is a non-invasive diagnostic tool widely used to assess brain function, particularly in the diagnosis and monitoring of epilepsy and encephalopathy. However, research on the EEG and seizure characteristics of pertussis encephalopathy in young infants was limited. This study was the first to retrospectively analyze the EEG features and seizure of pertussis encephalopathy in young infants. The findings of this study will help clinicians better understand the pertussis encephalopathy, enabling early identification and diagnosis.

### 1.1 Subjects and methods

#### 1.1.1 Subjects

This study included infants diagnosed with pertussis encephalopathy at Wuhan Children's Hospital between October 2022 and March 2024, a period coinciding with a local pertussis epidemic. A retrospective analysis was conducted on their series of EEG and clinical data. Patients were selected based on positive PCR detection of *Bordetella pertussis* in nasopharyngeal secretion samples using a testing kit produced by Shengxiang Biotechnology Co., Ltd. Patients with incomplete clinical data were excluded. In this study, according to the the literature pertussis encephalopathy was defined as the presence of acute cerebral dysfunction (e.g., altered mental status) during severe pertussis, after appropriate investigations to rule out other causes (9). The study was approved by the Medical Ethics Committee of Wuhan Children's Hospital (Approval No.: WU Paediatrics 2019013), and informed consent was obtained from the guardians of all infants.

#### 1.1.2 Methods

##### 1.1.2.1. Clinical data

Patient medical records were reviewed to collect the following data: gestational age, Apgar score, mode of delivery, perinatal complications, age at onset, gender, white blood cell count and its changes, body temperature, pertussis vaccination status, EEG background activity, EEG during electroclinical seizures (ECSzs) and electrographic seizures (ESzs), interictal EEG, laboratory test results, cranial magnetic resonance imaging (MRI) results, hospital stay and prognosis. Exclusion criteria: Patients with incomplete data or those who abandoned treatment.

##### 1.1.2.2 EEG examination

EEG signals were collected using a Nihon Kohden EEG system, with the average reference electrode as the reference. Nineteen recording electrodes were placed according to the international 10–20 system. The software filter was set to 0.5–70.0 Hz, and the EEG data were acquired at a sampling rate of 1,000 Hz. The monitoring duration was 2–4 h, including at least one complete wake-sleep-wake cycle. EEG interpretation was independently performed by two associate chief physicians in a blinded manner, and consensus was reached. EEG descriptions was reported using standardized critical care EEG terminology (ACNS Standardized Critical Care EEG Terminology 2021 version) (10). Seizure was broadly defined to include both ECSzs (events with both EEG seizure activity and accompanying clinical manifestations) and ESzs (events with clear EEG seizure activity but no observable clinical correlates). All seizures were identified based on EEG monitoring. We have explicitly defined the EEG criteria for seizure identification, which require evolution in frequency, morphology, and/or location and a minimum duration of ≥10 s. The term “migratory seizures” was defined as focal seizures with observed clinical and/or electroclinical migration between cerebral hemispheres.

## 2 Results

### 2.1 General information

Among the 6 cases, 1 were male and 5 were female. All cases were born at term without a history of intrauterine distress or asphyxia at birth, with an Apgar score of 9 or above. The age of onset ranged from 1 month and 16 days to 3 months and 13 days after birth, with an average age of onset of 2 months and 8 days. All cases were admitted to the intensive care unit due to “paroxysmal cough, dyspnea, with or without convulsions”. All cases underwent endotracheal intubation with ventilator-assisted breathing. All cases exhibited altered mental status, with three presenting as stupor and three as somnolence. The hospitalization duration for the six cases ranged from 30 to 47 days, with an average of 38.5 ± 5.68 days. None of the mothers of the children received pertussis vaccination during pregnancy. None of the cases had received pertussis vaccination. The highest white blood cell count during the course of the disease ranged from 39.49 to 81.09 × 10^9^/L, and the peak body temperature ranged from 37.9 to 39℃. None of the cases had a family history of febrile convulsions or epilepsy. Detailed patient information is shown in Table 1.

**Table 1:** Clinical information in 6 cases with pertussis encephalopathy.

Case	Gender	Age of onset	GA	Apgar score	Perinatal complications	PVS	Tmax (°C)	MFD	*θ*	Seizure^a^	Hospital stay (days)	Highest WBC count (10^9^/L)	Prognosis
1	F	2M16D	39W+2D	9	None	No	39	√	-	ECSz, ESz	47	51.15	Seizure-free, No lag
2	F	2M10D	39W+6D	10	None	No	39	√	√	ECSz, ESz	36	63.03	Seizure-free, No lag
3	F	3M13D	38W+6D	9	None	No	39	√	-	ECSz, ESz	37	81.09	Seizure-free, No lag
4	F	1M21D	39W+1D	9	None	No	37.9	√	√	ECSz, ESz	40	39.96	Seizure-free, No lag
5	F	1M16D	40W+2D	10	None	No	38	√	√	ECSz	30	44.19	Seizure-free, slightly lag
6	M	2M3D	39W+5D	9	None	No	38	√	√	ECSz	41	39.49	Seizure-free, significant lag

ECSz, electroclinical seizures; ESz, electrographic seizure; D, day; F, female; FS, focal seizure; GA, Gestational age; M, male; M, month; MFD, multifocal discharge; PVS, pertussis vaccination status; Tmax, peak body temperature during hospitalization; WBC, white blood cell; θ, θ rhythm.

√: Observed in the series of electroencephalograms.

-: Not observed in the series of electroencephalograms.

^a^ All electroclinical seizures in the children were confirmed by EEG.

### 2.2 Background and interictal EEG

**Figure 1:**
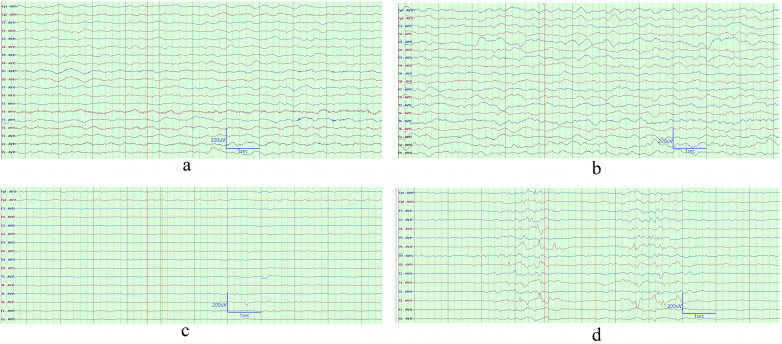
The background EEG activity of cases. **(a)** The recording from ICU day 2 in Case 2, during which the infant was stupor and on oxygen via nasal cannula (NC), demonstrated background activity characterized by Generalized Rhythmic Delta Activity (GRDA). **(b)** The recording from ICU day 6 in Case 4, during which the infant was somnolent, endotracheally intubated (ETI), and under sedation, demonstrated background activity characterized by GRDA. **(c)** This tracing represented the EEG of Case 2, performed on ICU day 16 while the infant was in a stupor, ETI, and under sedation, showing background activity suppression with excessive discontinuity during sleep in 2 cases. **(d)** This tracing represented the EEG of Case 2, performed on ICU day 16 while the infant was in a stupor, ETI, and under sedation, showing excessive discontinuity during sleep. The EEG recording parameters were set as follows: a sampling rate of 1,000 Hz, high-pass filter at 0.5 Hz, low-pass filter at 70 Hz, and sensitivity at 10 µV/mm. Certain channels were masked in the display due to unstable signals.

**Figure 2:**
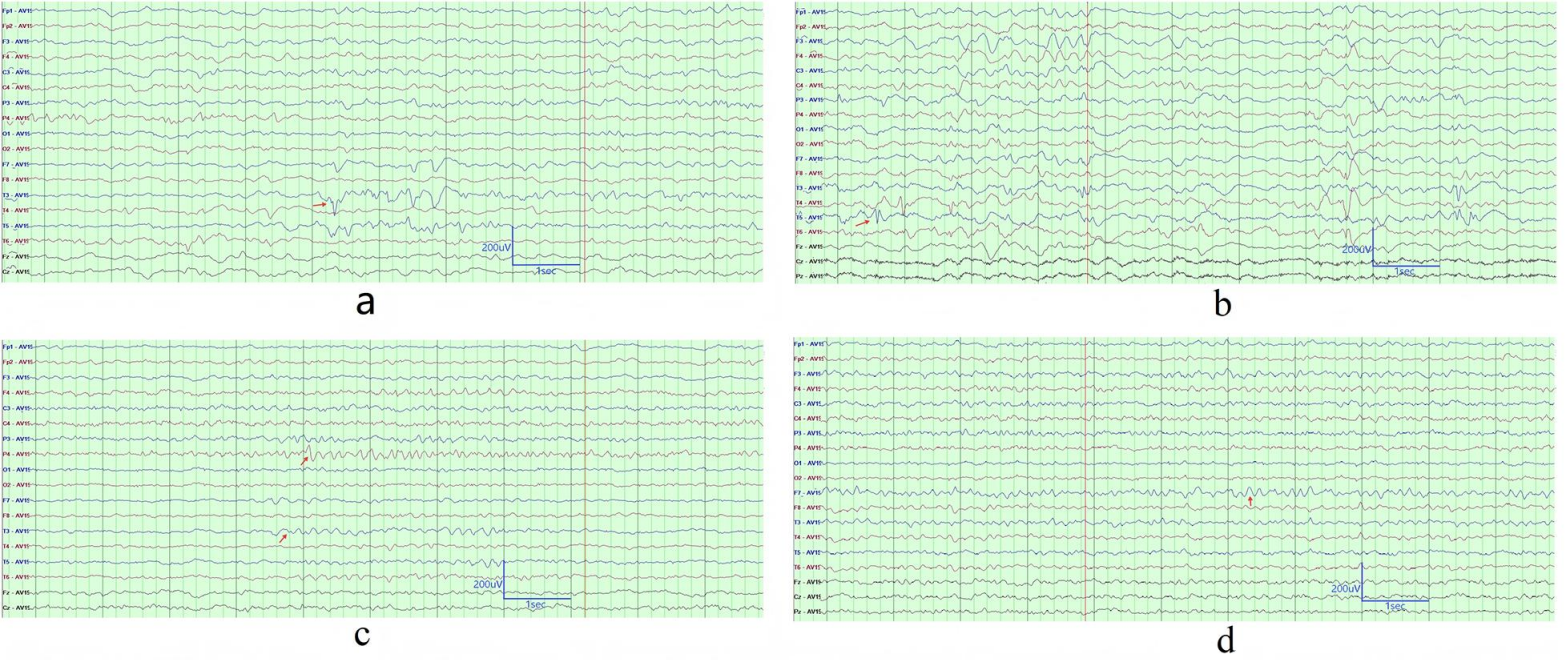
Interictal abnormal discharges and theta rhythm. **(a)** Prominent triphasic sharp and sharp-slow waves in the left temporal region were recorded, which was from the second EEG of Case 4 performed on ICU day 13. **(b)** Prominent triphasic sharp and sharp-slow waves in the bilateral temporal region were recorded, which was from the EEG of Case 6 performed on ICU day 20. **(c)** Prominent theta rhythm in the left temporal and right parietal regions were recorded, which was from the EEG of Case 4 performed on ICU day 13. **(d)** Prominent theta rhythm in the left temporal region were recorded, which was from the EEG of Case 6 performed on ICU day 15. The EEG recording parameters were set as follows: a sampling rate of 1,000 Hz, high-pass filter at 0.5 Hz, low-pass filter at 70 Hz, and sensitivity at 10 µV/mm. Certain channels were masked in the display due to unstable signals.

All cases with pertussis encephalopathy underwent 3–7 EEG examinations during their ICU hospitalization. Specifically, Case 1 and Case 2 respectively completed 6 sessions, Case 3 completed 7 sessions, Case 4 completed 3 sessions, Case 5 completed 4 sessions, and Case 6 completed 5 sessions. All serial EEG results were abnormal. Medium- to high-amplitude 2.5–3.5 Hz Generalized Rhythmic Delta Activity (GRDA) was detected in five cases. Notably, GRDA was recorded once in Cases 2, 3, and 5 without sedated endotracheal intubation, while all other cases were recorded under sedation with endotracheal intubation. (Figures 1a,b). Under sedation with endotracheal intubation and assisted ventilation, the background EEG activity in Case 1 and Case 2 exhibited background activity suppression with discontinuity during sleep in 2 cases (Figures 1c,d). All cases showed multifocal sharp waves and sharp-slow waves. In four cases, the phase of these abnormal waves occasionally consisted of three components, predominantly in the temporal region (Figures 2a,b). Four cases demonstrated focal theta rhythms. Among these, three showed prominence in the temporal region (Case 2, Case 4, and Case 5), and one (Case 6) exhibited a prominent and persistent theta rhythm in the left temporal region (Figures 2c,d). Four cases (Case 1, Case 2, Case 4, and Case 5) displayed alpha rhythms or beta rhythms. Table 2 summarized the case-by-case timeline of ICU days, clinical status, antiseizure medications, and key EEG findings. Cases 1 and 2 were transferred to our ICU after receiving 4 days and 1 day of ICU care at external hospitals, respectively. Therefore, the total duration of ICU stay for these two cases includes the days spent in both the external and our hospital ICUs.

**Table 2:** A timeline table days in ICU, clinical status, antiseizure medications, and key EEG findings from six cases.

Case	Days in ICU	clinical status	ASMs	Key EEG findings^a^
1	5^b^	Stupor, ETI, sedation	No	Frequent MFD, one FS (FC).
	13	Stupor, ETI, sedation	LEV	Frequent MFD, one ESz
	18	Stupor, ETI, sedation	LEV	Frequent MFD, several ESzs
	29	Stupor, ETI, sedation	LEV	Background activity suppression with discontinuity during sleep, frequent MFD, four ESzs
	35	Stupor, ETI, sedation	LEV, TPM	GRDA, frequent MFD, over ten FSs (BA, AUT)
2	2^c^	Stupor, O₂ via NC	No	GRDA, sharp-and-slow waves over the anterior regions（Occasional）
	16	Stupor, ETI, sedation	No	Background activity suppression with discontinuity during sleep, frequent MFD, frequent FSs (FC, BA, AUT) and ESzs
	22	Stupor, ETI, sedation	LEV, TPM	Abundant MFD, several ESzs.
3	3	Stupor, O₂ via NC	No	GRDA, frequent MFD
	12	Stupor, ETI, sedation	No	GRDA, frequent MFD, Several FSs (FC) and ESzs
	17\23	Stupor, ETI, sedation	LEV	GRDA, Abundant MFD, multiple FSs (FC, BA ).
4	6	Somnolence, ETI, sedation	No	GRDA, frequent MFD, two FSs (FC )
	13	Somnolence, ETI, sedation	LEV	GRDA, abundant MFD, several ESzs
5	9	Somnolence, ETI, sedation	No	Frequent MFD
	26	Somnolence, ETI, sedation	N0	GRDA, Frequent MFD, seven FSs (BA)
	33	Somnolence, O₂ via NC	LEV	GRDA, frequent MFD
6	1	Somnolence, ETI, sedation	No	Background activity suppression, Frequent MFD
	20	Somnolence, O₂ via NC	No	Abundant MFD, one FS (BA).

ASMs, Antiseizure medications; AUT, automatism; BA, behavioral arrest; EEG, Electroencephalogram; Electrographic seizure (ESz); ETI, endotracheal intubation; FC, focal clonus; FS, focal seizure; GRDA, Generalized Rhythmic Delta Activity; ICU, Intensive Care Unit; LEV, Levetiracetam; MFD, multifocal discharges; O₂via NC, oxygen via nasal cannula; TPM, Topiramate.

^a^ Although this table presented the majority of the findings from the 31 EEG examinations performed across all cases, no electroclinical or electrical seizures were detected in subsequent monitoring (results not shown).

^b^ ICU day 5 (including 4 days at referring hospital + 1 day post-transfer to our center).

^c^ ICU day 2 (including 1 days at referring hospital + 1 day post-transfer to our center).

### 2.3 Seizure manifestations and ictal EEG characteristics

**Figure 3:**
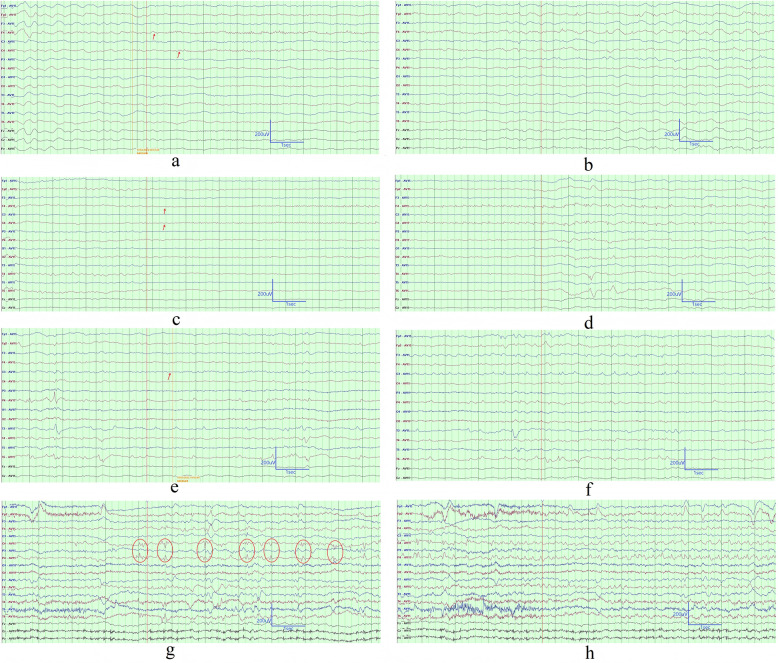
EEG showing electroclinical seizure onset and electrographic seizure onset. **(a,b)**. This continuous EEG tracing represents the onset of a focal seizure in Case 1 on ICU day 5, starting with low amplitude β rhythm in the right frontal and central regions, evolving into rhythmic sharp and slow waves, with a total duration of approximately 5 min. The complete EEG data for the seizure episode was provided in Supplementary Video S1. **(c,d)**. This continuous EEG tracing represents the onset of an electrographic seizure in Case 2 on ICU day 16, starting with low-amplitude beta rhythms in the right frontal and central regions, evolving into periodic low-amplitude sharp waves, with a total duration of approximately 2 min. **(e,f)**. This continuous EEG tracing represents the onset of a focal seizure in Case 2 on ICU day 16, starting with low amplitude α rhythm in the left central regions, evolving into periodic low amplitude sharp waves, with a total duration of approximately 9 min. The complete EEG data for the seizure episode was provided in Supplementary Video S2. **(g,h)**. This continuous EEG tracing represents the onset of an focal seizure in Case 6 on ICU day 20, starting with periodic low amplitude sharp waves in the right central and parietal regions, with a total duration of approximately 7 min. The EEG recording parameters were set as follows: a sampling rate of 1,000 Hz, high-pass filter at 0.5 Hz, low-pass filter at 70 Hz, and sensitivity at 10 µV/mm. Certain channels were masked in the display due to unstable signals.

Six cases were monitored for one or more electroclinical seizures (ECSz). Electrographic seizures (ESz) were recorded in Cases 1, 2, 3, and 4. Seizure onset involved three or more brain regions, including the frontal, central, parietal, and temporal areas in Case 1, Case 2, and Case 3. In Cases 1, 2, and 3, the ictal EEG onset was characterized by focal low-amplitude β or α rhythms, focal medium-amplitude slow-wave rhythms (2–3.5 Hz), or focal periodic sharp waves, followed by an electrographic evolution (in frequency, amplitude, and location) over a period of 2–20 min (Figures 3a–f, Supplementary Video S1,S2). In Case 4, seizure onset involved the left centro-parietal and right parietal regions, with ictal EEG showing focal 2–3.5 Hz slow-wave rhythms or focal low-amplitude beta rhythms, followed by an electrographic evolution (in frequency, amplitude, and location) over a period of 60 s to 2 min. In Case 5, seizure onset involved the left frontal and right central areas. The ictal EEG displayed focal periodic sharp waves, also followed by an electrographic evolution (in frequency, amplitude, and location) over a period of 6–9 min. In Case 6, seizure onset was localized to the right centro-parietal region. The ictal EEG exhibited focal periodic positive sharp waves, similarly followed by an electrographic evolution (in frequency, amplitude, and location) over a period of 5 min (Figures 3g,h). In Case 1 and Case 2, ECSz manifested as focal clonic movements, behavioral arrest, and automatisms (e.g., lip-smacking), with durations ranging from 2 to 20 min. In Case 3, ECSz presented with focal clonic movements and behavioral arrest, lasting from several seconds to 5 min and 30 s. In Case 4 and Case 6, ECSz were characterized by focal clonic movements, lasting 60–80 s in Case 4 and approximately 5 min in Case 6. In Case 5, ECSz manifested as behavioral arrest, with durations ranging from 6 to 9 min. Cases 1, 2, 3, and 6 exhibited migratory ECSz and ESz (Supplementary Video S3,S4). During the EEG monitoring, 4 cases (Case 1, 2, 3, and 5) exhibited 7 or more seizures. WBC count at the time of most frequent seizures was 8.48 × 10^9^/L, while peak WBC count during the illness was 51.15 × 10^9^/L in case 1. WBC count at the time of most frequent seizures was 14.35 × 10^9^/L, while peak WBC count during the illness was 63.03 × 10^9^/L in case 2. WBC count at the time of most frequent seizures was 9.21 × 10^9^/L, while peak WBC count during the illness was 81.09 × 10^9^/L in case 3. WBC count at the time of most frequent seizures was 11.26 × 10^9^/L,while peak WBC count during the illness was 44.19 × 10^9^/L in case 5.

### 2.4 White blood cell (WBC) counts during ICU admission

All cases presented with marked leukocytosis during the initial phase of ICU admission. The dynamic changes in white blood cell counts (x10⁹/L) during the ICU stay were as follows: Case 1 peaked on ICU day 5 (51.15) and gradually declined to 9.10 by ICU day 23. Case 2 (with pertussis encephalopathy) showed a characteristic rise-and-fall pattern, peaking on ICU day 3 (63.03) and decreasing to 11.75 by ICU day 16. Case 3 peaked on ICU day 2 (81.09), the highest value in the series, and fell to 8.07 by ICU day 16. Case 4 reached its peak on ICU day 4 (39.96) and declined to 9.03 by ICU day 13. Case 5, with an initial value of 44.19 on ICU day 1, decreased to 11.26 by ICU day 11. Case 6 was transferred to our hospital after successful resuscitation from an in-hospital cardiac arrest. The arrest occurred during outside treatment for a paroxysmal cough that had persisted for over 20 days, with significant worsening in the preceding 10 days. Following ICU admission, the patient exhibited a continuous downward trend in white blood cell count, which dropped from 32.21 on day 1 to 8.95 10⁹/L by day 6. Overall, the white blood cell counts demonstrated a gradual return toward normal levels with treatment progression.

### 2.5 Cranial imaging and cerebrospinal fluid results

**Figure 4:**
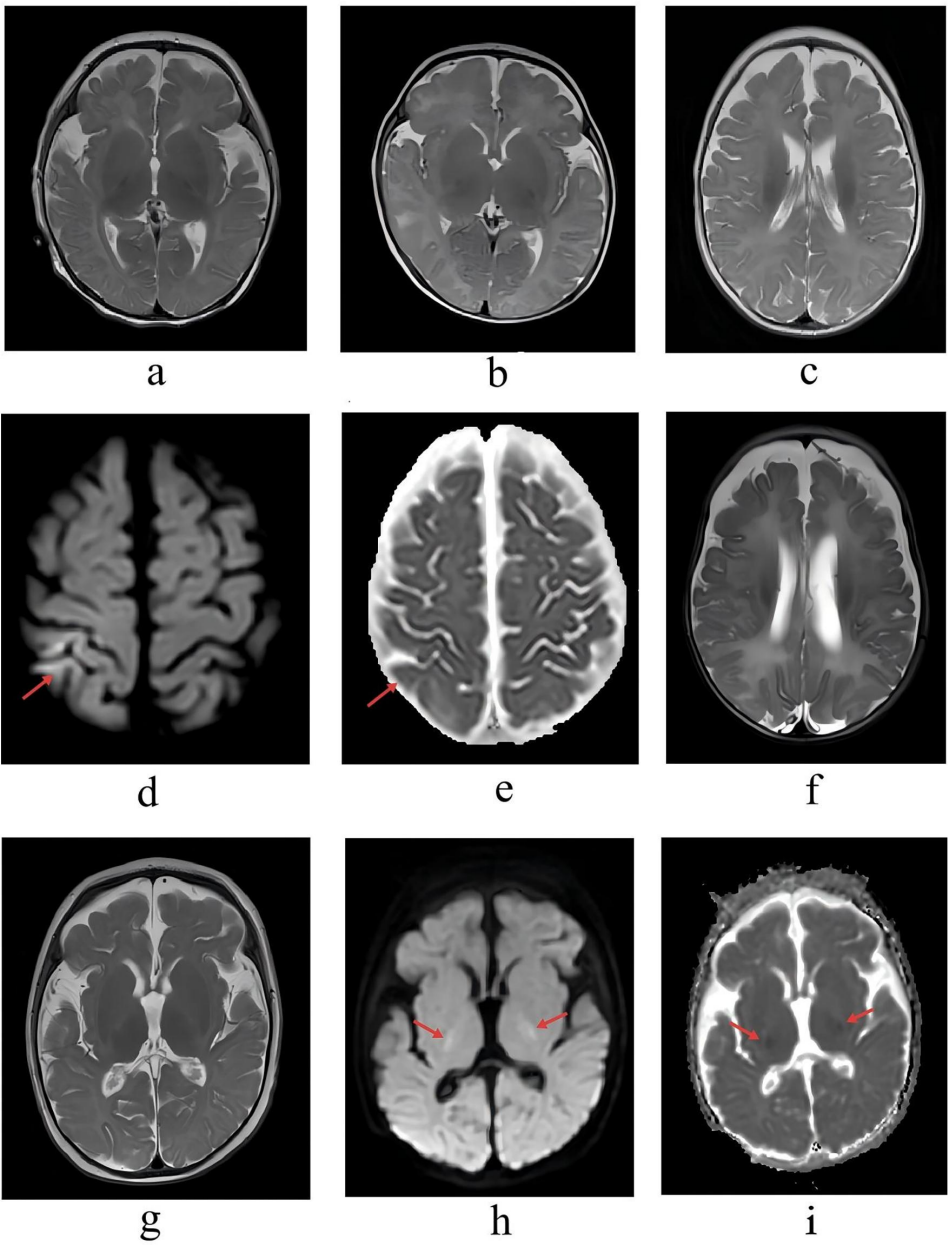
Cranial MRI (including DWI) findings during ICU admission. **(a,b)** Slight widening of frontal and temporal extracerebral spaces in Cases 1 and 2. **(c)** Mild bilateral ventricular enlargement, slight frontal extracerebral space widening, and slight corpus callosum thinning in Case 3. **(d,e)** Slight bilateral ventricular enlargement, with high DWI signal and corresponding ADC mild hypointensity in the right parietal lobe in Case 4. **(f)** Widened extracerebral spaces in bilateral frontal and temporal regions and the cerebral longitudinal fissure in Case 5. **(g–i)** Widened cerebral sulci and fissures, slightly high DWI signals in bilateral posterior limbs of internal capsules, and notably low ADC signal in Case 6.

All patients underwent non-contrast cranial MRI including Diffusion-Weighted Imaging (DWI) sequences during ICU treatment. Case 1 (ICU day 7) and Case 2 (ICU day 3) showed slight widening of extracerebral spaces in the frontal and temporal regions (Figures 4a,b). Case 3 (ICU day 8) exhibited mild bilateral ventricular enlargement, slightly widened frontal extracerebral spaces, and slight thinning of the corpus callosum (Figure 4c). Case 4 (ICU day 32) demonstrated mild bilateral ventricular enlargement and a hyperintense signal on DWI in the right parietal lobe, which appeared as a corresponding mild hypointense signal on ADC maps (Figures 4d,e). Case 5 (ICU day 15) showed widening of extracerebral spaces in bilateral frontal and temporal regions and the cerebral longitudinal fissure (Figure 4f). Case 6 (ICU day 18) presented widened cerebral sulci and fissures along with slightly hyperintense signals in the bilateral posterior limbs of the internal capsules (Figure 4g), with corresponding hypointensity on ADC maps confirming restricted diffusion; follow-up at 19 months demonstrated resolution of these hyperintensities and improvement of the sulcal/fissural widening (Figures 4h,i). Regarding cerebrospinal fluid (CSF) analysis, Case 1 (ICU day 20) showed protein 0.78 g/L with positive qualitative test; Case 2 (ICU day 3) had protein 0.68 g/L, chloride 106.80 mmol/L, and positive glucose qualitative test; Case 3 (ICU day 4) showed protein 0.94 g/L with positive glucose qualitative test; Case 4 (ICU day 7) had chloride 114.10 mmol/L and glucose 4.63 mmol/L (mildly elevated); Case 5 (ICU day 15) showed protein 0.95 g/L. The nucleated cell counts for these five cases were 1, 16, 1, 3, and 15 × 10⁶/L, and red blood cell (RBC) counts were 0, 0, 2,000, 0, and 2,000 × 10⁶/L respectively, with the elevated RBC counts of 2,000 × 10^6^/L in Cases 3 and 5 attributed to traumatic lumbar puncture. CSF analysis was not performed for Case 6.

### 2.6 Clinical outcomes and follow-up

The follow-up period for six patients ranged from 9 months to 2 years and 5 months. Their current ages ranged from 1 year to 2 years and 6 months. At 7 months of age, the EEG showed a normal background with occasional multifocal discharges during sleep in Case 1. The cases had not experienced seizures since discharge and showed no significant delays in cognitive or motor development. No seizures have occurred since discharge in Case 2, and cognitive and motor development were within normal limits. EEG at 10 months of age was normal in Case 3, no seizures have been reported post-discharge, and cognitive and motor development were on track. EEG at 4 months of age was normal in Case 4. The case had remained seizure-free since discharge, with no delays in cognitive or motor development. Following discharge, Case 5 had remained seizure-free and showed a neurodevelopmental profile of normal intellect despite mildly delayed gross motor skills. Notably, at 1 year of age, the infant can sit independently but had not achieved crawling. Since discharge, Case 6 had been seizure-free with no repeat EEG. At 13 months, examination revealed severe global developmental delay and prominent upper motor neuron signs, including generalized hypertonia, persistent fisting, and absent voluntary grasp.

## 3 Discussion

Currently, there was limited research both domestically and internationally on the seizures and EEG characteristics of pertussis encephalopathy in infants. This study is the first to report on these aspects.

Pertussis may lead to relatively serious complication such as pulmonary hypertension, heart failure, and encephalopathy (11). Studies have reported that severe pertussis mainly occurs in children under 3 months of age, with a mortality rate of 34.2% for severe pertussis, and the majority of deaths occurring in patients under 6 weeks of age (7). In our study, the onset age of severe pertussis ranged from 1 month and 16 days to 3 months and 13 days after birth, with an average onset age of 2 months and 8 days, consistent with previous research. This is mainly due to the immature immune system and thorax development in infants, narrow airways and weaker cough reflex, as well as delayed ciliary movement and mucous airway secretions caused by pertussis toxin, leading to airway obstruction and the development of severe pertussis (12). It has been reported that pertussis vaccination during pregnancy can effectively reduce the incidence of severe pertussis in infants (13). However, the Chinese Center for Disease Control and Prevention has not yet recommended routine pertussis vaccination for pregnant women. In this case report, none of the infants’ mothers had received the pertussis vaccine during pregnancy. The average hospitalization duration of 38.5 ± 5.68 days in our cases highlighted the significant burden of pertussis on healthcare resources, particularly in pediatric intensive care units. Prolonged hospital stays not only increase healthcare costs but also place emotional and financial strain on families. Therefore, early diagnosis, prompt initiation of appropriate treatment, and preventive measures such as vaccination for pregnant women were crucial in reducing the severity of the disease and minimizing hospitalization duration.

Children with pertussis exhibited several notable laboratory abnormalities, primarily marked leukocytosis (14). One study found that severe pertussis in infants under three months was more critical, with non-survivors showing significantly higher leukocyte counts (15). In our study, the white blood cell (WBC) of the infants were also markedly elevated, with peak values ranging from 39.49 to 81.09 × 10^9^/L, consistent with previous research (15). Pertussis encephalopathy is a rare but severe complication of pertussis, occurring in 0.5%–1% of all cases, but with a higher incidence in children under two years old (16). Based on the data from this study, WBC counts in infants with pertussis encephalopathy after ICU admission exhibited the following characteristics: peak levels were typically reached early in the disease course (usually within 1–5 days of ICU admission), followed by a progressive decline with treatment and disease progression, often returning to near-normal levels approximately 2–3 weeks after admission. Notably, at the time points when seizures were most frequently detected via EEG monitoring, the peripheral WBC counts of the patients (ranging from 8.48 to 14.35 × 10⁹/L) were significantly lower than their respective peak levels during the disease course. This suggested that the occurrence of seizures was not directly linearly correlated with the absolute WBC count, which indicated that the pathogenesis of neurological complications may involve more complex pathophysiological processes. This finding highlighted that during the treatment of severe pertussis, even when WBC counts have significantly decreased, clinicians must maintain a high level of vigilance for neurological complications and should not lower their guard against seizure risk based solely on the improvement of inflammatory markers.

This study found that seizures in infants with pertussis encephalopathy were exclusively focal in origin, often manifested with multifocal onset, and in some cases exhibited migratory patterns, suggesting the presence of multifocal cortical hyperexcitability. ECSz manifestations includeded localized clonic movements, behavioral arrest, and automatisms. Notably, behavioral arrest and automatisms in our cases were challenging to detect clinically, underscoring the critical role of EEG in evaluating seizure. It was noteworthy that, although the primary EEG examinations conducted in this study lasted 2–4 h, some cases exhibited seizures with prolonged duration (up to 5 min or even 20 min) and a significant increase in frequency. The migratory nature of these seizures may have directly contributed to the extension of individual seizure episodes. A prior study also reported a 10-year-old pertussis patient with focal status epilepticus (17). These findings that status epilepticus may occur in pertussis encephalopathy. The exact pathophysiological mechanisms of pertussis encephalopathy remain unclear. Proposed hypotheses include central nervous system hemorrhage or hypoxia due to coughing-induced elevated venous pressure, vascular occlusion/venous congestion from lymphocyte emboli, hypoglycemia, exacerbation of unrecognized underlying neurological conditions, and effects of *Bordetella pertussis* toxins—though the pathogen has never been isolated from cerebrospinal fluid (18, 19).

Since cerebrospinal fluid examination and imaging manifestations of severe pertussis encephalopathy in infants lack specificity (20), the diagnosis is typically based on clinical symptoms and risk factors. In the study, the main imaging features included mild or marked atrophic-like changes.In one case, MRI performed on the 32nd day of ICU admission showed a hyperintense signal in the right parietal lobe on DWI with corresponding mild hypointensity on ADC maps, indicating possible localized cytotoxic edema. In another case, DWI performed on the 18th day of ICU admission revealed symmetrical hyperintense signals in the bilateral posterior limbs of the internal capsules with corresponding ADC hypointensity, also suggesting edematous changes. Combined with literature reports of findings such as caudate nucleus enhancement (21). These observations collectively suggested that MRI in pertussis encephalopathy may manifest both focal diffusion restriction and atrophic-like changes. However, all imaging changes observed in this study lacked disease specificity, and similar manifestations were commonly seen in other infectious, metabolic, or hypoxic-ischemic brain injuries. It was important to note that this study had several inherent limitations due to its retrospective design. Most critically, we were unable to precisely determine the exact timing of the clinical diagnosis of “pertussis encephalopathy” for each case or accurately quantify the interval between the onset of neurological symptoms and the MRI examination. This limitation hindered a deeper interpretation of the pathophysiological mechanisms underlying the imaging findings.

Regarding CSF analysis, this study performed biochemical and routine examinations on five cases. Mildly elevated protein levels (0.68–0.95 g/L) were observed in four cases, mildly reduced chloride levels in three cases, and mildly elevated glucose in one case. Notably, nucleated cell counts in all five cases showed no significant increase (1–16 × 10⁶/L). These findings further indicated that CSF alterations in pertussis encephalopathy may lack specificity. Although mildly decreased CSF chloride was not a characteristic feature of pertussis encephalopathy, considering the clinical profile of pertussis, we speculated that its mechanism may be related to fluid and electrolyte disturbances caused by the disease. It should be noted that this retrospective study has certain limitations: CS*F* testing was mostly conducted during the early phase of the disease (ICU days 3–20), and most patients underwent only a single test, making it difficult to dynamically observe the evolution of CSF indicators.

In the series of EEG examinations conducted in this study, GRDA was observed in five cases. Notably, GRDA was recorded once in Cases 2, 3, and 5 without sedated endotracheal intubation, while all other cases were recorded under sedation with endotracheal intubation. Additionally, two cases in the study exhibited background activity suppression with discontinuity during sleep under sedation. A notable limitation of our retrospective study was the lack of precise data on sedative dosages (Midazolam). Although the use of Midazolam can alter the characteristics of EEG background activity and even induce burst-suppression patterns, the observation of GRDA in three cases without sedation still may support the conclusion that pertussis encephalopathy itself can lead to GRDA. Additionally, all cases in this study exhibited multifocal sharp waves and sharp-slow waves. Among them, four patients presented with triphasic sharp waves or sharp-slow waves in the temporal region, while four cases showed a significant increase in theta rhythm activity in the temporal region. These findings collectively suggest that the temporal lobe may have a higher susceptibility or vulnerability to involvement in pertussis encephalopathy. However, this observation still requires validation through larger-sample studies, and the underlying pathophysiological mechanisms warrant further in-depth investigation.

The neurological symptoms of pertussis encephalopathy can be severe (22). In this study, all cases experienced no further seizures after discharge. Four cases showed no significant delays in motor or intellectual development, one case was slightly behind in motor development, and 1 case was significantly behind in motor cognitive development. These results suggested that the neurological prognosis of infants with pertussis encephalopathy varies greatly. In our study, the case with significantly delayed motor cognitive development demonstrated specific MRI findings, with involvement of the bilateral internal capsule posterior limbs and markedly widened cerebral sulci and fissures. It is hypothesized that the widened cerebral sulci and fissures may be attributed to cerebral hypoxia resulting from severe pertussis, with consequent developmental impairment. At the same time, his EEG showed a prominent and relatively persistent theta rhythm in the left temporal region. Whether these abnormal indicators were related to a poor prognosis and whether they were risk factors for a poor prognosis still require further research with a large amount of data. The limitations of this study include its single-center retrospective design and the small number of severe pertussis encephalopathy cases collected, which may introduce bias into the conclusions. Therefore, more cases are needed in the future to further analyze the clinical characteristics and ictal EEG features of this condition.

In summary, infantile pertussis encephalopathy was characterized by electroclinical-electrographic seizures. These seizures were focal and may have a multifocal or migratory onset, manifesting as focal clonic seizures, behavioral arrest, or automatisms. Corresponding EEG may show GRDA, multifocal sharp/sharp-slow waves (sometimes triphasic), and temporal-dominant theta rhythms. A notable clinical observation was the lack of correlation between seizure burden and WBC count variations. Despite the potential for achieving seizure control through intensive therapy, long-term neurodevelopmental outcomes demonstrate significant heterogeneity.

## Data Availability

The original contributions presented in the study are included in the article/Supplementary Material, further inquiries can be directed to the corresponding author/s.

## Supplementary material

Supplementary Video 1 Full EEG progression of one ECSz in Case 1 (EEG correlates of Fig. 1a, b).

Supplementary Video 2 Full EEG progression of one ECSz in Case 2 (EEG correlates of Fig. 1e, f).

Supplementary Video 3 **Migrating focal seizure in Case 1. **This video presents the full 8-minute 50-second EEG of a migrating focal seizure in Case 1 (recorded on ICU day 35). The scalp EEG demonstrated the characteristic migration of the ictal focus: originating in the right mid-posterior temporal region, shifting to the left mid-posterior temporal region. The synchronized clinical video showed the corresponding behavioral manifestation, featuring pronounced hypoactivity and oral automatisms (lip-smacking).

Supplementary Video 4 **Migrating focal seizure in Case 2. **This video presented a 13-minute ECSz from Case 2 (recorded on ICU day 16). The ictal EEG onset was localized to the right fronto-central region, with subsequent migration to the left centrotemporal region,and finally to the right region. The clinical correlate during this episode consisted of rhythmic limb jerking.
